# Autologous Hematopoietic Stem Cell Transplantation (aHSCT) in a 56-Year-Old Filipino Female With Relapsing and Remitting Multiple Sclerosis: A Case Report

**DOI:** 10.7759/cureus.87558

**Published:** 2025-07-08

**Authors:** Jon Stewart H Dy, Pamela Marie M Cope, Cymbeline Perez-Santiago, Francisco F Lopez

**Affiliations:** 1 Neurosciences, St. Luke's Medical Center College of Medicine, Quezon, PHL; 2 Neurosciences, St. Luke's Medical Center, Manila, PHL; 3 Neurosciences, St. Luke's Medical Center, Taguig, PHL; 4 Internal Medicine, Hematology, Oncology, St. Luke's Medical Center, Taguig, PHL

**Keywords:** ahsct, disease modifying therapies, edss, multiple sclerosis, rituximab

## Abstract

Multiple sclerosis (MS) is the most common autoimmune demyelinating disease of the central nervous system. Treatment options include oral, subcutaneous, and intravenous disease-modifying therapies (DMT). An alternative therapy in the treatment of MS is autologous hematopoietic stem cell transplantation (aHSCT). It has been shown to benefit patients with relapsing and remitting multiple sclerosis (RRMS) who have an inadequate response to DMTs. In this case report, we present the first local documentation of a 56-year-old Filipino female diagnosed with RRMS who underwent aHSCT. Prior to treatment, she was maintained on rituximab for two years and had an expanded disability status scale (EDSS) score of one. Due to the development of new-onset first-degree atrioventricular (AV) block, she was subsequently switched to aHSCT and underwent the procedure without any complications. Two years after treatment, she has not had any relapses, is not on any DMT, has a stable EDSS score of one, and is independent in all her activities of daily living. Our case underscores the utility of considering aHSCT as a possible cost-effective, safe, and effective treatment option for Filipino patients with RRMS.

## Introduction

Multiple sclerosis (MS) is the most common autoimmune demyelinating disorder of the central nervous system [1]. Diagnosis is established through the 2024 revised McDonald's criteria with the use of clinical features, magnetic resonance imaging (MRI), and cerebrospinal fluid (CSF) findings [2]. The mainstay of treatment for MS is the use of disease-modifying therapies (DMTs). To date, there are no curative treatments, and there is no single therapeutic target for MS. The goal of treatment is to achieve disease remission by reducing inflammation, limiting primary demyelination and secondary axonopathy, and preventing relapses. DMTs have been shown to be efficacious in patients with relapsing and remitting multiple sclerosis (RRMS). Treatment options include oral, subcutaneous, and intravenous (IV) therapies [3]. The decision to switch between DMTs is made if there are new clinical relapses, an increase in MRI lesion size or number, or worsening disability after at least one year of treatment on a DMT [3]. Autologous hematopoietic stem cell transplantation (aHSCT) is an alternative therapeutic option that has been shown to benefit patients with RRMS who have an inadequate response to DMTs. The risk-benefit profile for patients with RRMS who will undergo such therapy should consider the age, disease phase (early or late), and disability status through the expanded disability status scale (EDSS) [3]. 

To date, there has been no local documentation of a Filipino patient with MS who underwent aHSCT. In this study, we report the case of a middle-aged Filipino female diagnosed with RRMS who has been undergoing biannual rituximab infusion and eventually underwent aHSCT without periprocedural and postprocedural complications. She is the first local documentation of a Filipino patient with RRMS who underwent aHSCT.

## Case presentation

**Figure 1:**
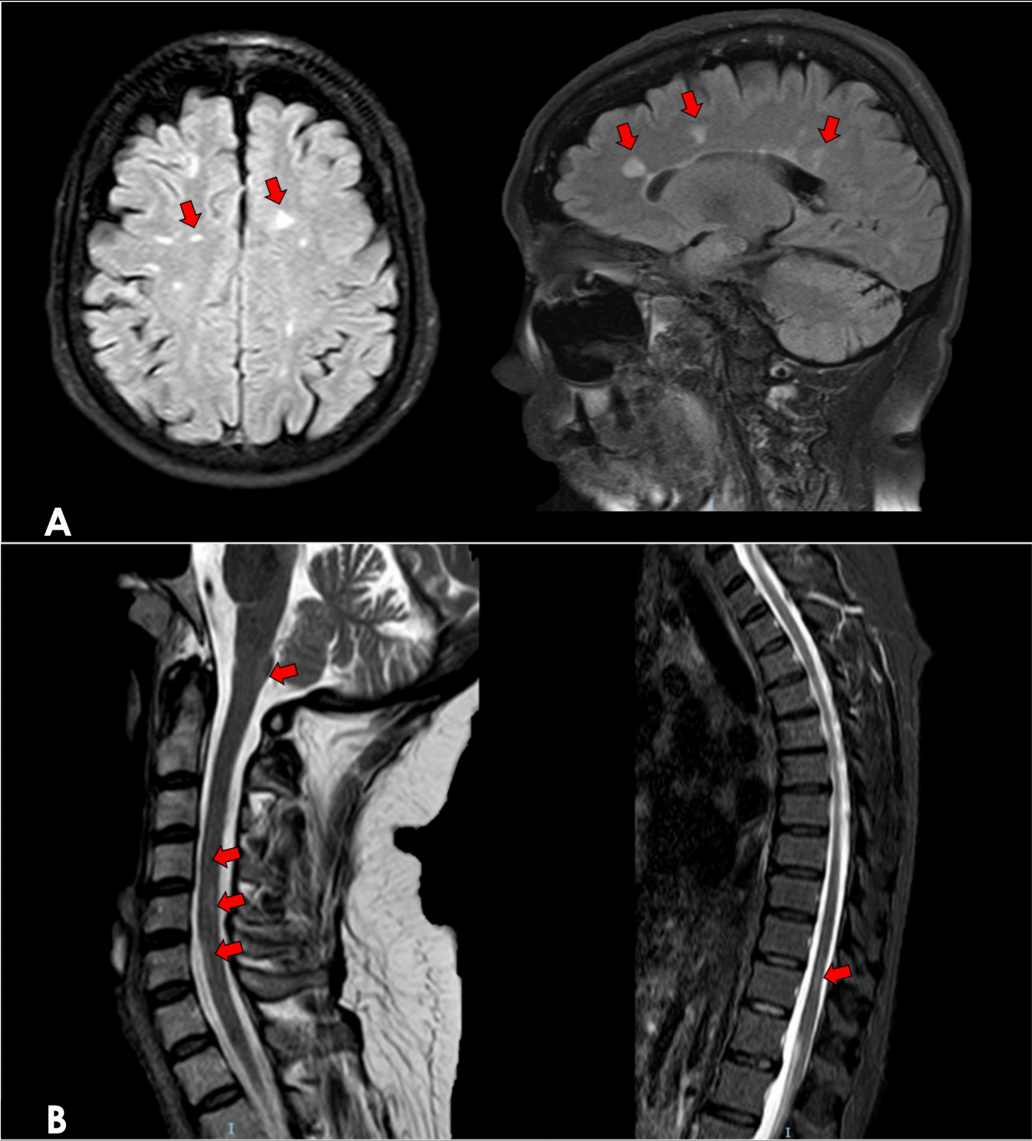
MRI done two months prior to aHSCT (February 2023) (A) Representative axial and sagittal brain MRI images show multiple FLAIR hyperintense lesions over the juxtacortical, subcortical, and periventricular areas with mild global cerebral atrophy. (B) Representative sagittal spinal MRI images show T2 hyperintense lesions over the posterior cervicomedullary junction, cervical spinal cord (C4-C5 and C6-C7), and thoracic spinal cord (T10-T11) with mild cord atrophy. In comparison to previous brain and spinal MRI procedures, these findings are stable and show no radiologic progression. aHSCT - autologous hematopoietic stem cell transplantation; FLAIR - fluid-attenuated inversion recovery

Our patient is a 56-year-old Filipino female with controlled hypertension and prediabetes who was diagnosed with RRMS in 1996 when she was 28 years old. Her initial clinical symptoms included asymmetric weakness of all limbs along with sensory deficits over the head and the chest, for which she was given pulse methylprednisolone therapy on initial diagnosis and was not maintained on any DMT thereafter. The rest of her past medical history is unremarkable, and there is no heredofamilial history of autoimmune or demyelinating diseases. She continued to have relapses but without any disability progression 25 years after the diagnosis, which prompted the initiation of rituximab infusions (500mg per infusion) for a total of four cycles given every six months from 2021 to 2022. She did not have any relapses, achieving no evidence of disease activity (NEDA)-three (Figure 1) while on rituximab, and she remained at EDSS one with right medial rectus paresis and neuropathic pain over both hands and feet with no cognitive deficits and no other focal neurological deficits. A therapeutic decision was made to switch to aHSCT from rituximab due to the development of new-onset first-degree atrioventricular (AV) block, with the goal of achieving long-term disease remission without the continued need for a maintenance DMT. Ocrelizumab was not yet locally available during this time. 

Baseline laboratory examination (Table 1), urinalysis, and chest radiograph results were unremarkable. To mobilize her hematopoietic stem cells, she was given granulocyte-colony stimulating factor (G-CSF) at a dose of 10 micrograms (mcg) per kilogram (kg) subcutaneously daily for five days. During her admission for aHSCT in April 2023, she was given pre-induction chemotherapy with carmustine, cytarabine, etoposide, melphalan, and horse anti-thymocyte globulin (ATG). Thereafter, 3.9 million cluster of differentiation 34 positive (CD34+) stem cells were infused. There were no periprocedural complications or relapses. She was given prophylactic antibiotics (co-amoxiclav, metronidazole, trimethoprim-sulfamethoxazole), antiviral (valacyclovir), and antifungal (fluconazole) medications before the procedure. Post procedure, she was treated with meropenem for febrile neutropenia. The rest of her in-hospital admission was unremarkable, and she was eventually discharged stable after 21 days of hospital stay.

**Table 1:** Baseline laboratory tests prior to aHSCT g/dL - grams per deciliter; mm3 - cubic millimeters; fL - femtoliter; mg/dL - milligrams per deciliter; mmol/L - millimoles per liter; U/L - units per liter; aHSCT - autologous hematopoietic stem cell transplantation

Test	Results	Reference value
Hemoglobin	12.0 g/dL	11.6-15.5 g/dL
Hematocrit	37.4%	36.0-47.0%
White blood cell cunt	4,340 mm3	4800-10,800 mm3
Neutrophils	71%	40-74%
Lymphocytes	17%	19-48%
Monocytes	9%	3-9%
Platelet count	455,000/mm3	150,000-400,000/mm3
Mean corpuscular volume	93 fL	82-98 fL
Mean corpuscular hematocrit	30 fL	28-33 fL
Mean corpuscular hemoglobin concentration	32%	32-38%
Creatinine	0.51 mg/dL	0.55-1.2 mg/dL
Blood urea nitrogen	8 mg/dL	9-23 mg/dL
Sodium	139 mmol/L	136-145 mmol/L
Potassium	4.1 mmol/L	3.5-5.1 mmol/L
Magnesium	2.0 mmol/L	1.6-2.6 mmol/L
Chloride	103 mmol/L	98-107 mmol/L
Bicarbonate	29.0 mmol/L	20.0-31.0 mmol/L
Alanine transferase	33 U/L	10-49 U/L
Aspartate transferase	26 U/L	0-34 U/L
Albumin	4.4	3.2-4.8 g/dL

Follow-up of our patient two years after aHSCT shows no relapses (NEDA-three), no disability progression (EDSS one), and she is currently not maintained on any DMT. She is currently on regular annual surveillance monitoring with her attending neurologist. 

## Discussion

MS is an autoimmune and demyelinating disease that commonly affects young adults with an average age of onset between 20 and 50 years of age and a female-to-male ratio of 3:1. Increased prevalence and incidence of MS are seen in areas farther from the equator. It is associated with various host factors, agent factors, and environmental factors. The most common symptoms of MS include fatigue, sensory and motor deficits, vision loss, gait difficulty, and cognitive impairment [1]. Currently, there are no locally available epidemiologic studies on MS. In our institution, a total of 243 autoimmune and demyelinating diseases were documented in 2023, the most common of which was multiple sclerosis. 

Patients with MS should be presented with all reasonable DMTs, taking into consideration their comorbidities, disease severity, medication adverse effects, and treatment adherence. In the Philippines, the only available DMTs for MS include fingolimod, rituximab, and ocrelizumab (Table 2). These are available only in private tertiary centers, and only Rituximab is included in the Philippine National Formulary Essential Medicines List [3]. Fingolimod is an orally available S1P receptor analog administered at 0.5 milligrams (mg) per day, requires baseline ophthalmologic evaluation, and cardiac monitoring is needed when administering the first dose. Rituximab is a chimeric anti-CD20 monoclonal antibody administered at 500mg intravenously every cycle, with the first two doses given two weeks apart and subsequent doses given every six months. This requires screening for heart blocks and arrhythmias, tuberculosis, and hepatitis B due to the risk of reactivation. Ocrelizumab is a fully-humanized anti-CD20 monoclonal antibody administered at 300mg intravenously every cycle, with the same dosing regimen as rituximab. There are currently no established guidelines regarding the duration of use of the aforementioned DMTs, hence continued administration of DMTs such as rituximab similar to our patient requires surveillance monitoring to prevent the long-term risks associated with medication-specific adverse effects and prolonged immunosuppression, including reactivation of latent infection (tuberculosis, hepatitis B, progressive multifocal leukoencephalopathy) and secondary malignancies [4, 5]. Clinicians must also take into account that the effectiveness of DMTs directed at active disease activity diminishes over time in patients with sustained absence of further disease activity [5]. Finally, compliance with oral and IV medications is a real-world issue for Filipino patients, which warrants further study of alternative therapeutic options.

**Table 2:** Available DMTs in the Philippines and Their Local Cost PHP = Philippine Pesos; $ = US Dollar *Drug prices listed as of June 13, 2025 [6, 7, 8]

DMTs	Cost (Per Dose)	Cost (5 year duration)
Fingolimod	PHP 336 (~$5-$6) per 500mcg tablet	PHP 604,800 (~$10,800)
Rituximab	PHP 40,757 (~$700-$725) per 500mg vial	PHP 407,570 (~$7,300)
Ocrelizumab	PHP 150,000 (~$2,600-$2,700) per 300mg vial	PHP 1,500,000 (~$26,600)

The aim of aHSCT is to reset the immune system in patients with MS. Candidates for aHSCT should ideally satisfy the following criteria: <50 years of age, RRMS (with active disease activity), course of the disease <10 years, EDSS >2 but </= 5.5 (those who are ambulatory but with worsening disability), breakthrough disease activity (clinical and radiologic) despite treatment with high efficacy DMTs, or those who are unable to tolerate DMTs. The risks associated with the procedure include relapse of MS during preparation prior to the procedure and adverse effects from the conditioning regimen. The risk-benefit ratio is favourable during the early phase of the disease, when the patient is young and not disabled, as in our case. During this stage, benefit from the procedure is expected due to suppression of inflammatory activity, with stabilisation or improvement of disability. High risks and minimal benefit would be expected in the late stages of the disease, in which the procedure should no longer be recommended. Lastly, the risk-benefit ratio of the procedure for patients in the intermediate stage is more uncertain, and establishing the indication for treatment is more difficult [9].

Evidence supporting the safety, efficacy, and superiority of aHSCT has been increasing in recent years. A meta-analysis study showed a 2.1% procedure-related mortality, 17.1% two-year and 23.3% five-year progression rate, with 83% and 67% showing no evidence of disease activity at two years and five years, respectively [10]. Direct comparisons between aHSCT and other available DMTs have been done. The Autologous Stem-cell Transplantation International MS (ASTIMS) trial comparing aHSCT and Mitoxantrone showed a lower annual relapse rate [11]. The Multiple Interventional Study of Transplantation (MIST) trial comparing DMTs (excluding ocrelizumab and alemtuzumab) showed a lower rate of progression of disease at five years post-transplantation [12]. A study comparing aHSCT and alemtuzumab showed that both treatments had comparable efficacy over a five-year follow-up period [13]. A comparative effectiveness study showed superiority of aHSCT over fingolimod, cladribine, and alemtuzumab, and marginal superiority over natalizumab in preventing relapses and recovery from previously accrued disability [14]. A cohort study showed superior efficacy of aHSCT compared with alemtuzumab and ocrelizumab over a five-year follow-up period [15]. A prospective study showed improved symptom severity with aHSCT over the course of two years [16]. In contrast, a study showed that aHSCT can improve outcomes in those with progressive forms of multiple sclerosis, but it does not halt progression [17]. A quality-of-life study showed that aHSCT is associated with a clinically meaningful improvement in the quality of life, with most occurring early within the first year following the procedure [18]. A limitation of existing literature is the lack of studies supporting the safety and efficacy of aHSCT with a longer follow-up period (>5 years). Hence, ongoing randomized clinical trials further exploring its long-term efficacy compared with other immune reconstitution therapies are underway.

To date, there are currently no locally available studies documenting the epidemiology of MS, the safety and efficacy of aHSCT, and studies comparing it against locally available DMTs. A local study showed that the Philippines faces significant challenges in terms of the availability of accurate epidemiologic information, resource allocation, access to, and provision of diagnostic and treatment services [19]. The predominant mode of payment for healthcare in the country is an out-of-pocket system [20]. This presents us with challenges in starting DMTs for our Filipino patients with MS, including issues related to treatment compliance. The local cost of aHSCT in our institution is PHP 1,500,000 to PHP 2,000,000 (~$26,000 to $36,000). Given this treatment landscape, Filipino patients would opt for cost-effective, safe, and efficacious treatment options that would not require long-term maintenance treatment on a DMT. In this regard, aHSCT presents to our patients an attractive therapeutic option. Our study, therefore, highlights the challenges of treatment access and compliance with DMTs, and the utility of presenting alternative therapeutic options for Filipino patients with MS with active disease activity and worsening disability.

Our study has several limitations. First, it is a single case study that can only suggest the benefit of aHSCT in Filipino patients with RRMS. Further higher-powered studies with larger sample sizes are needed before establishing its overall efficacy and safety in Filipino patients. Second, despite the more economical local cost of the procedure, it is available in only the major tertiary centers in the Philippines. Lastly, there are no standard guidelines or referral pathways for patients with MS to undergo aHSCT. Therefore, future studies must also aim to construct and establish a proper guide for clinicians on when and where to refer Filipino patients with MS who are ideal candidates for aHSCT.

## Conclusions

In summary, we presented the first local documentation of a 56-year-old Filipino female with RRMS who underwent aHSCT. This case highlights the importance of considering alternative therapeutic options in patients with MS who have active disease activity. While the safety and efficacy of the procedure have been extensively studied in recent years, the long-term clinical outcomes post-procedure remain unclear. Future studies are needed to analyze its cost-effectiveness, safety, and long-term efficacy in Filipino patients with MS, thereby controlling disease activity and reducing morbidity and mortality associated with progressive disease. 
